# Gene Therapy Tools for Diseases Caused by Mutations of the Mitochondrial Genome

**DOI:** 10.3390/ijms27125517

**Published:** 2026-06-18

**Authors:** Vladislav Simonov, Sergey Rastorguev

**Affiliations:** Center for High-Precision Technologies for Biomedicine, Pirogov Russian National Research Medical University, Ostrovityanova 1, 117513 Moscow, Russia; rastorgueff@list.ru

**Keywords:** mitochondrial manipulation, gene therapy, mtDNA, allotopic expression, mitoCRISPR, mitoTALEN, mitoZFN, DdCBE

## Abstract

Mitochondrial DNA (mtDNA) mutations are associated with a diverse spectrum of diseases and pose a significant threat to human health. Despite their importance as therapeutic targets, the unique structural and electrochemical properties of mitochondria—most notably the impermeable inner mitochondrial membrane and the high membrane potential—present formidable challenges for the targeted delivery of therapeutic agents. Currently, there are no approved curative treatments for patients harboring pathogenic mtDNA mutations. In this review, we discuss recent advancements in gene therapy for mitochondrial genome-related disorders, with a particular focus on allotopic expression of mtDNA-encoded genes and mitochondrial genome editing technologies. We conclude that allotopic expression currently stands as the most promising approach for near-term clinical implementation. But we also pay great attention to programmable nucleases and base editors utilizing RNA-independent DNA recognition which are evolving with remarkable speed.

## 1. Introduction

Mitochondria are semi-autonomous organelles originating from ancient endosymbiotic proteobacteria, whose primary function is the production of adenosine triphosphate (ATP), the usable form of energy for cells. The vast majority of mitochondrial proteins are encoded by the nuclear genome, translated in the cytosol, and imported into the mitochondrion. However, there is also a mitochondrial genome. It is circular and 16.5 kb in length. It encodes 22 tRNA genes, two ribosomal RNAs (rRNA), and 13 protein products. Of the 13 core subunits of the respiratory complexes encoded by the reduced mitochondrial genome, seven polypeptides are subunits of complex I, one is part of complex III, three are subunits of complex IV, and two are part of ATP synthase [1]. As these 13 proteins are core subunits of the OxPhos chain, any disruption to the structure, stability, or function of these subunits can have severe biochemical and physiological consequences [2]. Approximately 1 in 5000 individuals suffer from mitochondrial disease—a heterogeneous group of conditions caused by mutations in any of the >1400 genes encoding components of the organelle responsible for aerobic respiration [3,4,5]. Unlike the diploid nuclear genome, mitochondrial DNA is polyploid, with hundreds to thousands of copies per cell. This often leads to the coexistence of mutant and wild-type genomes within the same cell—a condition known as heteroplasmy [6]. The presence of wild-type genomes compensates for the effect of mutant genomes until the percentage of mutant mtDNA exceeds a certain threshold, after which clinical symptoms manifest [7,8]. Mitochondrial diseases most commonly affect tissues requiring high levels of cellular ATP for proper functioning, such as the nervous system, heart, skeletal muscles, and liver. Phenotypes, onset, and severity range from mild symptoms, such as exercise intolerance, to neonatal lethality [9]. Mitochondrial genome mutations most commonly cause mitochondrial encephalomyopathy, lactic acidosis, and stroke-like episodes (MELAS), myoclonic epilepsy with ragged red fibers (MERRFs), Neurogenic muscle weakness, ataxia, and retinitis pigmentosa (NARP), and Leber hereditary optic neuropathy (LHON). LHON causes optic nerve atrophy, sudden loss of central vision, optic disc edema, and large central visual field defects. Patients with MELAS may experience episodic sudden headaches, intermittent atypical migraines, and intractable seizures. Patients with MERRF exhibit myoclonic epilepsy, ataxia, spasticity, generalized seizures, optic nerve atrophy, and dementia. They may also present with muscle weakness, myopathy, neuropathy, or sensorineural hearing loss. Neurogenic muscle weakness, ataxia, and retinitis pigmentosa NARP can represent a milder form of maternally inherited Leigh syndrome. Patients with NARP may present with developmental delay, dementia, seizures, ataxia, retinitis pigmentosa, proximal neurogenic muscle weakness, and sensory neuropathy [5]. Some of the most common mutations in the mitochondrial genome and the diseases they cause are listed in Table 1.

We explore the most promising strategies developed, discussing their mechanisms of action and potential for clinical application with the ultimate goal of providing a comprehensive understanding of the potential of gene therapy in treating mitochondrial diseases.

## 2. Allotopic Expression

The synthesis of mitochondrial DNA (mtDNA)-encoded proteins within the cytosol, a process known as allotopic expression, offers a compelling strategy for the genetic treatment of human diseases caused by mutations in the mitochondrial genome. The fundamental concept involves integrating a wild-type copy of a mutated mitochondrial gene into the nuclear genome, followed by the import of the resulting protein products into the mitochondria from the cytosol. This approach requires two critical modifications: the addition of a mitochondrial targeting sequence (MTS) to the gene to facilitate the translocation of the precursor protein into the mitochondrial matrix, and codon optimization to account for the divergence between the nuclear and mitochondrial genetic codes.

However, experimental attempts using various MTS and mtDNA-encoded genes in human cells have shown that precursor polypeptides are not consistently imported with high efficiency (the various MTS used for allotopic expression are listed in Table 2). Consequently, the repertoire of mtDNA-encoded polypeptides that can be successfully expressed and integrated into the mitochondrial respiratory chain complexes remains limited [23]. This constraint is primarily attributed to the high hydrophobicity of mtDNA-encoded proteins, which possess transmembrane domains that impede mitochondrial import. Precursors synthesized in the cytosol may fail to maintain an import-competent conformation necessary for efficient translocation. It is now widely recognized that the extreme hydrophobicity of mitochondrial-encoded proteins represents a formidable barrier to their mitochondrial import during cytosolic translation [24].

Currently, the most prevalent strategy involves the codon optimization of mitochondrial genes for nuclear translation, fused with an MTS derived from a nuclear-encoded mitochondrial protein. For instance, O’Connor et al. utilized patient-derived cybrid cell lines harboring a single point mutation in the overlapping region of the *ATP8* and *ATP6* genes. This mutation results in the loss of ATP8 expression and a reduction in ATP6 levels. Nuclear expression of ATP8 utilizing the ATP5G1 MTS restored ATP synthesis levels but failed to recover ATP hydrolysis. Notably, the co-expression of both *ATP8* and *ATP6* under similar conditions led to stable protein expression, successful integration into Complex V, and functional phenotypic rescue [25]. Similarly, Manfredi et al. reported the restoration of ATP synthesis in cybrids homoplasmic for the 8993T>G mutation following the allotopic expression of a codon-optimized ATP6 [26].

Furthermore, an optimized allotopic approach for ND4 has demonstrated success in clinical trials for Leber Hereditary Optic Neuropathy (LHON) [27,28], although it remains the only instance of successful clinical translation for allotopic mitochondrial gene therapy to date. In animal models, Begelman et al. achieved functional ATP synthase restoration in C57BL/6J(mtFVB) mice, which harbor a natural polymorphism (m.7778 G>T) in the *ATP8* gene, through allotopic expression [29]. Successful allotopic expression of ND3 in patient-derived cells has also been reported by Borna et al. [30].

A significant potential obstacle in expression of genes encoding highly hydrophobic proteins is their propensity for aggregation within the cytoplasm or on the mitochondrial outer membrane [31,32]. Oca-Cossio et al. reported that allotopically expressed apocytochrome b and ND4 failed to colocalize with mitochondria; instead, upon overexpression, they aggregated into cytoplasmic fibrils containing tubulin and vimentin [33]. Perales-Clemente et al. similarly observed that the allotopic expression of *ND6* led to cytoplasmic protein accumulation, although the extent of aggregation was not explicitly detailed [34]. Comparable effects have been documented during the allotopic expression of *Cox2* in yeast [35]. In such cases, allotopic expression not only fails to restore respiratory function but may also trigger the loss of mitochondrial membrane potential, ultimately leading to cell death [33]. Another challenge relates to hydrophobic proteins are potentially misdirected to the endoplasmic reticulum (ER) rather than the mitochondria [36]. Protein translocation to the ER occurs co-translationally and is initiated by the signal recognition particle (SRP), which binds to hydrophobic segments such as transmembrane domains or signal sequences [37]. This aberrant targeting from the mitochondria to the ER may be driven by increased hydrophobicity of either the C-terminal [38] or N-terminal [39] domains, a reduction in the net positive charge of the C-terminal domain [39,40], or specific post-translational modifications [41]. Knöringer et al. demonstrated that when mitochondrial protein translocation is blocked, mitochondrial membrane proteins tend to accumulate within the ER [42]. It is highly probable that the similarity and potential interchangeability of mitochondrial and ER-targeting signals possess significant adaptive value during cellular stress, as they serve to localize protein aggregation to a limited number of compartments. Finally, experimental evidence has shown that nuclear-expressed Cox1, Cytb, and ATP6 proteins predominantly colocalize with the ER [36,43].

Notably, in mammalian cells, many mRNAs are localized to the mitochondrial surface to facilitate the co-translational import of the resulting proteins [44]. Matsumoto et al. established that human mRNAs encoding highly hydrophobic proteins with more than two transmembrane domains are typically associated with outer membrane-bound polysomes. In contrast, mRNAs encoding proteins with one or no transmembrane domains are distributed between both mitochondrial-associated and cytosolic polysome fractions [45]. In this regard, it is hypothesized that the 3′-untranslated region (3′UTR), by ensuring mRNA localization to the mitochondrial surface, may significantly facilitate protein import. For instance, *ATP6*, *ND4*, and *ND1* constructs fused with the 3′UTR of the *COX10* gene encoded proteins that exhibited markedly improved mitochondrial entry [46]. Kaltimbacher et al. further confirmed the beneficial effect of the 3′UTR from the nuclear-encoded *SOD2* gene on the import of ATP6 [47]. However, a subsequent study by Chin et al., which evaluated 31 MTS and 15 3′UTRs for their ability to localize allotopically expressed proteins, concluded that the choice of MTS is the primary determinant of successful optimization, while 3′UTR optimization exerts negligible influence [48].

**Table 2:** Signals of mitochondrial localization, which were used for alotopic expression of mitochondrial genome genes.

Mitochondrial Target Signal	Source Protein	Source Protein UniProt ID	Grand Average of Hydropathy (GRAVY Scope)	References	Result
ATP5MC1	ATP5MC1 *Homo sapiens*	P05496	0.641	[25,49]	Mainly successful
ATP6	ATP6 *Chlamydomonas reinhardtii*	Q8H762	0.533	[50]	unsuccessful
Cox3	Cox3 *Chlamydomonas reinhardtii*	Q9FV97	0.224	[50]	unsuccessful
COX8A	COX8A *Homo sapiens*	P10176	0.349	[33,34,36]	unsuccessful
COX10	COX10 *Homo sapiens*	Q12887	0.128	[27,46]	Mainly successful, but it is possible that a successful transfer requires 3′UTR of COX10

Phase 3 clinical trials of lenadogene nolparvovec, a gene therapy product based on the allotopic expression of the ND4 gene, have demonstrated sustained bilateral visual improvement and a favorable safety profile at a 5-year long-term follow-up [51]. Additionally, a clinical study targeting the mutated ND1 gene has been registered, although its findings remain unpublished to date [52,53]. Regrettably, lenadogene nolparvovec currently stands as the only allotopic expression-based therapy in late-stage clinical development.

The unique physiological characteristics of the eye—including its immune privilege, relative anatomical isolation allowing for direct vector delivery (e.g., via intravitreal or subretinal injection), small volume, and the feasibility of non-invasive longitudinal monitoring—position ocular diseases as a primary frontier for innovative biotechnological solutions. These include tetracycline-induced expression, polycistronic vectors [54], and the utilize of dual-AAV systems [55]. The potential clinical success of lenadogene nolparvovec may serve as a critical milestone, paving the way for gene therapies targeting more complex organs, such as the brain and skeletal muscles.

## 3. Xenotopic Expression

Xenotopic expression refers to the cytosolic expression of a gene orthologous to a mitochondrial gene but derived from a different species, followed by the import of the protein into the mitochondria to functionally replace a mutant counterpart [56,57]. Throughout evolution, orthologs of several genes encoded by the human mitochondrial genome have been successfully transferred to the nucleus in other organisms [58]. Such genes typically possess an endogenous MTS and encode less hydrophobic proteins; however, they are often found in phylogenetically distant taxa, such as algae [59,60] and higher plants [61]. This evolutionary distance raises concerns regarding the efficiency with which these xenotopic products can integrate into human OXPHOS complexes.

Unique mitochondrial genomic architectures are characteristic of green algae. For instance, in *Chlamydomonas reinhardtii* and *Polytomella* sp., the *ATP6* [62] and *COX3* [63] genes are nuclear-encoded, while *COX2* has not only been transferred to the nucleus but also split into two subunits, cox2a and cox2b [64]. As early as 2004, González-Halphen et al. proposed leveraging these unique evolutionary features to develop gene therapies for human mitochondrial diseases [65]. Initial attempts using human codon-optimized *COX3* and *ATP6* fused with *C. reinhardtii* MTS demonstrated successful mitochondrial translocation in human cells but failed to achieve integration into the OXPHOS complexes. This failure was potentially attributed to the introduction of specific amino acid substitutions intended to reduce protein hydrophobicity [50]. In contrast, the expression of full-length ATP6 from *C. reinhardtii* in human cells harboring pathogenic ATP6 mutations resulted in successful mitochondrial import, integration into human ATP synthase, and the restoration of ATP synthesis [66].

Furthermore, while *Saccharomyces cerevisiae* mitochondria lack a classical respiratory Complex I, they utilize NADH dehydrogenases (Nde1, Nde2, and Ndi1) to catalyze electron transfer from NADH to ubiquinone without proton translocation [67]. The application of NDI1 in a mouse model of Leber Hereditary Optic Neuropathy led to a 1.5-fold reduction in retinal ganglion cell (RGC) mortality and a 1.4-fold decrease in optic nerve atrophy, resulting in significant preservation of retinal function [68]. NDI1 has also been shown to improve mitochondrial function and attenuate reactive oxygen species (ROS) levels in mouse models of glaucoma [69] and in cell culture models of Parkinson’s disease, although it does not prevent the formation of Lewy bodies. Moreover, this approach improved behavioral outcomes and biochemical parameters in a murine model of Parkinson’s disease [70].

## 4. Strategies for Correcting Mitochondrial tRNA Mutations

All tRNAs required for the human mitochondrial translation machinery are encoded by mtDNA. Pathogenic point mutations frequently occur within these mitochondrial tRNA (mt-tRNA) genes [71]. Given that mitochondrial RNA import is generally highly inefficient in humans, a direct allotopic approach—involving the nuclear expression of mt-tRNAs—is considered unfeasible. Nevertheless, several strategies have been proposed to partially restore the intra-mitochondrial aminoacyl-tRNA pools.

While most organisms synthesize their mitochondrial translation components exclusively within the organelle, *Saccharomyces cerevisiae* possesses a cytosolic lysine tRNA (tRNA-Lys) that is partially imported into the mitochondria [72]. Kolesnikova et al. demonstrated that this yeast tRNA can also be imported into human cell cultures harboring the m.8344A>G mutation (*MT-TK*), where it partially restores mitochondrial function [73]. Building on this yeast tRNA scaffold, Karicheva et al. engineered leucyl-tRNA variants designed for cytosolic synthesis and subsequent mitochondrial translocation. However, they were unable to identify a variant that simultaneously possessed both high recognition determinants for leucyl-tRNA synthetase and efficient import signals; consequently, only limited functional recovery was achieved in m.3243A>G (MT-TL1) mutant cells [74].

In 2012, Wang et al. reported the successful suppression of *MT-TK* and *MT-TL1* mutations using a hybrid tRNA containing a stem-loop sequence from H1 RNA and the 3′UTR of the human mitochondrial ribosomal protein S12 (MRPS12) gene. They also claimed to achieve mitochondrial import of COX2 mRNA using the same targeting sequences. However, these results remain controversial due to a lack of independent replication and ongoing debates regarding the intrinsic importability of H1 RNA (discussed further in the “CRISPR/Cas9 and Other RNA-Guided Editors” section) [75].

An alternative approach involves the RNA Import Complex (RIC) from *Leishmania tropica.* Unlike humans, Kinetoplastids have lost all mitochondrial tRNA genes during evolution and rely on the multi-subunit RIC to import tRNAs from the cytoplasm [76,77]. It has been shown that the Leishmania RIC can enter human cells via a caveolin-1-dependent pathway, where it facilitates the import of endogenous cytosolic tRNAs (including tRNA-Lys) and restores mitochondrial function in MT-TK mutant cybrids [78,79]. Nonetheless, the requirement of nine distinct proteins for RIC assembly poses a significant challenge for in vivo therapeutic delivery.

Furthermore, Sieber et al. explored non-selective RNA transport using the RNA-binding protein DHFR fused to the MTS of *Neurospora crassa* ATP9. They achieved the delivery of approximately 5% of cytoplasmic tRNA-Ala into isolated mitochondria [80]. However, the efficacy of this artificial transporter has not been validated in mammalian cell cultures or animal models. Moreover, since DHFR binds RNA non-specifically, it likely transports various cytosolic RNAs up to 800 nucleotides in length. Such an influx of exogenous RNA could potentially interfere with mitochondrial matrix processes, such as replication inhibition [81].

In myoblast cultures derived from patients with the m.3243A>G mutation, respiratory function was partially rescued by overexpressing translation factors EFTu or EFG2, though no effect was observed with EFT or EFG1 [82]. Additionally, the τm5U-modifying enzyme MTO1 has been shown to restore mitochondrial activity in myoblasts from patients with MELAS and MERRF syndromes [83].

A substantial body of research has focused on the overexpression of amino-acyl-tRNA synthetases (ARSs). It is hypothesized that variations in endogenous ARS expression levels may contribute to the phenotypic diversity observed in individuals carrying pathogenic mt-tRNA mutations [84]. For instance, Perli et al. described a family patients harboring the pathogenic 4277T>C mutation in mt-tRNA(Ile), where the severity of clinical symptoms was found to correlate with the expression levels of the isoleucyl-tRNA synthetase gene, *IARS2* [85]. Furthermore, the overexpression of cognate aminoacyl-tRNA synthetases has been successfully employed to rescue mitochondrial dysfunction in various models carrying mutations in their respective mitochondrial tRNA genes [86,87,88,89].

Moreover, the deleterious effects of the m.3243A>G mutation are suppressed not only by the full-length leucyl-tRNA synthetase but also by its carboxy-terminal domain (Cterm) [90]. While the Cterm enhances de novo mitochondrial protein synthesis, it lacks a catalytic site, possesses no enzymatic activity, and does not influence the aminoacylation of mt-tRNA(Leu) [91]. Notably, as demonstrated by d’Amati et al., a 16-amino acid peptide derived from the Cterm is sufficient to improve the cellular phenotype compromised by the m.3243A>G and m.8344A>G mt-tRNA mutations [92]. In contrast, an analysis of the suppressive activity of mitochondrial alanyl-tRNA synthetase (AARS2) on mt-tRNA(Ala) revealed that *AARS2* overexpression restores a healthy phenotype by increasing aminoacylation activity rather than by stabilizing the tRNA structure [88]. These divergent mechanisms of action between leucyl-tRNA synthetase [93] and alanyl-tRNA synthetase [88] may be attributed to their classification into different enzyme classes [94,95]. Furthermore, aminoacyl-tRNA synthetases within the same functional group exhibit partial interchangeability in their capacity to mitigate the effects of tRNA mutations. Specifically, three human mitochondrial aminoacyl-tRNA synthetases—leucyl-, valyl-, and isoleucyl-tRNA synthetase—have been shown to enhance both the viability and bioenergetic profile of human transmitochondrial cybrid cells harboring pathogenic mutations in the mt-tRNA(Ile) gene [90]. The various approaches related to the expression of protein-coding genes, which were discussed in Section 2, Section 3 and Section 4, are listed in Table 3.

## 5. DNA Delivery to Mitochondria

While the MITO-Porter system can deliver RNA molecules up to 500 bp with moderate efficiency [97], the translocation of large DNA fragments, such as full-length plasmids, across the mitochondrial membrane remains a significant challenge with extremely low efficiency [98]. Nevertheless, several pioneering studies have reported successful mitochondrial transformation. Yasuzaki et al. utilized hydrodynamic injection to deliver the pHSP-mtLuc (CGG) vector, which contains a luciferase gene under the control of the mitochondrial heavy-strand promoter (HSP), resulting in detectable luciferase expression [99]. Similarly, Yamada et al. employed a MITO-Porter system modified with the KALA peptide to deliver pCMV-mtLuc (CGG) plasmids into the mitochondria, achieving luciferase expression driven by the CMV promoter [100]. Furthermore, Lyrawati et al. reported the delivery of pmtGFP—a self-replicating plasmid harboring the GFP gene—into macrophage mitochondria using dequalinium vesicles (DQAsomes) [101]. A notable advancement in viral-mediated delivery was achieved by Yu et al., who fused the adeno-associated virus (AAV) VP2 capsid protein with a mitochondrial targeting sequence (MTS) to deliver the *ND4* gene. The expression of wild-type (WT) *ND4* in cells carrying the 11778G>A mutation successfully restored defective ATP synthesis. Moreover, in vivo administration into rodent eyes resulted in transgene expression across most inner retinal neurons, effectively preventing vision loss and optic nerve atrophy [99]. The purported ability of mitochondria to maintain AAV sequences as episomes is particularly striking, as wild-type viruses do not naturally form episomes within this organelle. Although definitive evidence that mammalian mitochondria can support stable episomes is lacking, recombinant AAV (rAAV) may potentially integrate into the mitochondrial genome at a low frequency [102]. However, despite being first reported in 2012, this technology has not yet transitioned into routine laboratory practice and remains largely confined to its laboratory of origin. Consequently, the feasibility of AAV-mediated mitochondrial DNA delivery necessitates further independent validation and robust replication.

## 6. CRISPR/Cas9 and Other RNA-Guided Editors

The CRISPR/Cas9 system is a cornerstone of nuclear DNA editing, used for both generating mutations and correcting pathogenic alleles. The first report of successful mitochondrial DNA (mtDNA) editing was published by Jo et al. [103]. While several subsequent studies have claimed successful mtDNA correction using CRISPR/Cas9 [104,105], these findings are countered by numerous negative reports, with the primary bottleneck being the low efficiency of guide RNA (gRNA) translocation into the mitochondrial matrix [106]. A fundamental distinction between nuclear and mitochondrial CRISPR/Cas9 applications lies in the DNA repair mechanisms. Double-strand break (DSB) repair is notoriously inefficient in mammalian mitochondria [107,108]. Consequently, CRISPR/Cas9-mediated mtDNA editing likely functions not through precise repair, but through the selective degradation of mutant genomes, thereby modulating heteroplasmy levels. This mechanism is supported by observations of significant mtDNA copy number depletion following CRISPR/Cas9 treatment [109,110]. Therefore, the introduction of novel mutations (e.g., insertions or transversions) into the mitochondrial genome remains largely unfeasible. Moreover, this degradative approach poses a clinical risk for patients with high heteroplasmy levels (exceeding 85–90%), such as those with the m.8344A>G (*MT-TK*) mutation, where further depletion of mtDNA copies could exacerbate mitochondrial deficiency [111].

The targeting of CRISPR/Cas9 components to the mitochondria presents distinct challenges. While wild-type Cas9 lacks intrinsic tropism for eukaryotic organelles, it can be redirected on the nucleus or mitochondria using a targeting sequence [112,113]. However, the delivery of single-guide RNA (sgRNA) is far more complex. Unlike nuclear editing, where sgRNAs are transcribed in the nucleus and remain there, mitochondrial transport of RNA is highly selective and its mechanisms remain a subject of intense debate [114,115].

There is currently no consensus on the translocation machinery or the specific transcripts capable of entering the mitochondria. For instance, it was long believed that mammalian mitochondrial RNase P required a nuclear-encoded catalytic RNA subunit [116,117]. However, subsequent structural studies revealed that human mtRNase P is a protein-only complex (comprising three subunits) devoid of an RNA component [118]. This discovery casts significant doubt on strategies using the RP-loop for mitochondrial RNA targeting [119,120]. Similarly, RNase MRP, initially described as a mitochondrial ribonucleoprotein complex [121,122], has been shown to localize predominantly in the nucleolus, where it facilitates pre-ribosomal RNA processing [121,122,123].

Conflicting reports also exist regarding the mitochondrial transport of telomerase RNA (TERC) [124,125] and some nuclear-encoded long non-coding RNAs, as like as SAMMSON, for which an effect on mitochondrial function has been shown, which is why they are sometimes considered imported into the mitochondria, but no evidence has been provided [126]. In many cases, these RNAs may influence mitochondrial physiology indirectly—either via cytosolic RNA-binding proteins [127] or by regulating nuclear-encoded mitochondrial genes [128]—rather than through direct translocation. These indirect effects likely explain the lack of reproducibility in several RNA transport studies. Nonetheless, it is widely accepted that specific microRNAs (miRNAs) [129,130,131] and potentially some lncRNAs [132,133] are present in the mitochondrial matrix. Their transport may involve proteins such as Ago2 [134,135] and PNPase [135,136]. It is hypothesized that sgRNAs might utilize these same endogenous miRNA import pathways, which notably lack defined targeting sequences.

The limitations of RNA import can potentially be circumvented by delivering CRISPR/Cas9 components inside lipid nanoparticles (LNPs) [104]; however, the in vivo efficacy of this delivery strategy remains to be fully elucidated.

Intriguingly, Jo et al. reported that Cas9 exhibits an inherent tropism for mitochondria both in the absence and presence of a COX8A-derived MTS, and that gRNAs could translocate into the mitochondrial matrix without specific targeting signals [103]. Similar findings were shared by Wang et al. [105] and Bi et al. [110], who successfully employed SaCas9 with a COX8A MTS and sgRNAs lacking targeting sequences. However, these reports have been met with significant skepticism, most notably from Gammage et al. [137], who argue that spontaneous sgRNA transport is highly improbable given the extreme selectivity of the mitochondrial RNA import machinery [115].

In response to these challenges, subsequent studies have increasingly incorporated targeting signals derived from nuclear-encoded RNAs that are putatively imported into mitochondria. For instance, Schmiderer et al. failed to detect any editing effects using Cas9 and Cas9-BE3 even when employing the RP-loop [106]. Conversely, Hussain et al. successfully edited an ND4 mutation by fusing the sgRNA with an RP-loop derived from RNase P [109]. It is important to note, however, that the RNA component of RNase P, from which the RP-loop was derived, is now considered non-exported according to recent evidence [118], which casts doubt on the mechanism by which it might enhance sgRNA import. More recently, a hairpin structure from the long non-coding RNA RP11-46H11.3 was reported to possess high mitochondrial tropism; when fused with Cas9-sgRNA, it achieved a heteroplasmy shift comparable to that of a mito-TALEN targeting the same locus [138].

As an alternative to Cas9, the Cas12a (Cpf1) system has been proposed, which is claimed to target mitochondria more efficiently due to its requirement for shorter guide RNAs (crRNAs: ~40 nucleotides vs. sgRNAs: ~100 nucleotides) [139,140]. Nikitchina et al. demonstrated that short crRNAs for AsCas12a effectively penetrate the mitochondrial matrix independently of targeting hairpin sequences. Furthermore, Antón et al. highlighted the mitochondrial toxicity of SpCas9 fused with a COX8A MTS, which resulted in poor localization, impaired mitochondrial morphology, and functional decline in transiently transfected mammalian cells. In contrast, LbCas12a showed successful expression and localization within the mitochondrial matrix using several MTS (with Su9 being the most effective), although prolonged expression also induced mitochondrial damage. The authors suggest that the superior performance of LbCas12a may be due to its smaller size, higher positive charge, and lower hydrophobicity compared to SpCas9. Paradoxically, despite testing several sgRNA/crRNA versions with various targeting signals (FD, AD, or RP), Antón et al. were unable to definitively detect RNA entry into the matrix, and LbCas12a editing unexpectedly resulted in an increase in mtDNA copy number [139].

A novel approach recently proposed by Rimskaya et al. involves the use of Argonaute proteins from the mesophilic bacterium *Alteromonas macleodii* (AmAgo). Argonautes are significantly smaller than Cas-family nucleases (AmAgo is ~65 kDa, compared to ~160 kDa for Cas9 and ~150 kDa for Cas12a), which may facilitate their mitochondrial import. Furthermore, while Cas nucleases rely on long guide RNAs and specific protospacer adjacent motifs (PAMs), prokaryotic Argonautes (pAgos) utilize short single-stranded RNA or DNA guides (typically 16–20 nt) and operate independently of PAM sequences, offering greater targeting flexibility. However, the presence of endogenous miRNAs within the mitochondria remains a potential source of unintended off-target effects [141].

## 7. RNA-Independent Genome Editing Using Targetable Nucleases

Initially, the use of restriction endonucleases (REs) was proposed for mitochondrial genome cleavage. As previously discussed, the objective of this nuclease-based approach is to cleave a specific mtDNA locus present in only one haplotype. Since double-strand breaks (DSBs) almost invariably lead to the degradation of the mitochondrial genome in mammalian cells, the selective elimination of mutant mtDNA results in a proportional increase in wild-type genomes within the total population—a process known as a heteroplasmy shift. Although off-target DSBs should be strictly avoided due to potential adverse effects, the multi-copy nature of mtDNA renders non-specific breaks less catastrophic for the mitochondrial compartment than for the nuclear genome [107,108].

The primary limitation of this approach is that naturally occurring restriction endonucleases are not easily programmable. Consequently, the only relevant application involves mutations that spontaneously generate a recognition site for an existing RE. For instance, the m.8399G mutation in the MT-ATP6 gene, which causes Leigh syn-drome/NARP, creates a unique SmaI recognition site that is otherwise absent in the human and murine mitochondrial genomes. This approach has demonstrated high efficacy both ex vivo in cultured cells and in vivo in heteroplasmic mouse models [142]. However, the rarity of such mutations precludes this method from being applied to the vast majority of mitochondrial pathogenic variants.

Massive expansion of mitochondrial editing capabilities became possible with the advent of programmable nucleases, starting with Zinc Finger Nucleases (ZFNs). Zinc fingers, engineered to recognize specific trinucleotides, are typically linked in arrays of 3 to 6 modules to extend the target sequence. This modularity allows for the creation of ZFNs with bespoke DNA-binding specificity. To achieve targeted cleavage, the ZF binding domain is fused to the catalytic domain of the FokI nuclease. The FokI enzyme possesses distinct DNA-binding and cleavage domains, enabling the use of a non-specific catalytic domain to introduce breaks at predefined sites. To enhance specificity, the FokI nuclease (originally active as a homodimer) was engineered into an obligate heterodimer that forms active complexes only when bound to adjacent DNA sequences in an inverted orientation. MitoZFNs have been successfully used to edit several mutations, including m.5024C>T [143], m.8993T>G [144], and the CD4977 “common deletion” [144].

An alternative approach utilizes Transcription Activator-Like Effectors (TALEs) combined with the same FokI catalytic domain. These platforms, originally discovered in the proteobacteria Xanthomonas, were among the first versatile gene-editing tools [145]. The DNA-binding domain of TALEs comprises a modular and highly repetitive region where 33–35 consecutive amino acids (TALE repeats) possess specific residues at positions 12 and 13, known as Repeat Variable Di-residues (RVDs). TALENs were adapted for mitochondrial DNA (mitoTALENs) by incorporating an MTS, a specialized TALE DNA-binding domain, and the obligate heterodimeric FokI cleavage domain. This system has successfully shifted heteroplasmy for the m.5024C>T [146], m.3243A>G [147], and m.8344A>G [148] mutations. Notably, for reasons that remain not fully understood, some mutations could be successfully edited with ZFNs but not with TALENs [149]. Conversely, TALENs are generally considered to exhibit significantly lower off-target activity compared to ZFNs [150].

A major shared disadvantage of both systems is their dimeric architecture and the substantial size of the DNA-binding domains, which limits their compatibility with delivery vectors of restricted packaging capacity, such as adeno-associated viruses (AAV). To circumvent this limitation, Precision Biosciences developed the mitoARCUS system, based on the I-CreI meganuclease derived from the *Chlamydomonas reinhardtii* chloroplast genome. Unlike the large, dimeric ZFNs and TALENs, ARCUS operates as a monomer. Its coding sequence spans approximately 1100 bp, facilitating efficient packaging into AAV vectors. This engineered endonuclease was initially developed to target the m.3243A>G mutation, though it can potentially be reprogrammed to recognize and cleave a wide array of DNA sequences [151]. Recently, Bacman et al. utilized mitoARCUS to edit the m.5024C>T mutation (mt-tRNA(Ala) in murine skeletal muscle. The tool was administered via intramuscular injection of lipid nanoparticle complexes. Transient expression of mitoARCUS in the *tibialis anterior* (TA) muscle led to a substantial reduction in the mtDNA mutation load, which persisted for up to 42 weeks post-injection. In this model, the depleted level of mt-tRNA(Ala) serves as a molecular marker of mitochondrial dysfunction; notably, these levels were significantly restored in the treated muscles. Furthermore, in situ muscle force assessments following repetitive stimulation revealed that fatigability was markedly reduced in the treated TA muscle. These results demonstrate that transient expression of mitoARCUS via LNP/mRNA intramuscular injections exerts a sustained positive impact on muscles afflicted by mitochondrial myopathy [152]. Nevertheless, ARCUS targeting remains a significant challenge, as proper positioning of the active site requires the central region of the target sequence to consist of a pyrimidine followed by a purine [153]. This requirement somewhat constrains the selection of viable target sequences within the mitochondrial genome.

## 8. Mitochondrial Base Editors

In 2020, a breakthrough was achieved with the introduction of DdCBE (DddA-derived cytosine base editor) technology, the first tool capable of precise mitochondrial base editing. This system is based on the catalytic domain of the DddA deaminase from the Gram-negative bacterium *Burkholderia cenocepacia*. DddA exhibits deaminase activity toward cytosine, specifically targeting 5′-TC contexts, thereby inducing C-to-T transitions in double-stranded DNA. To mitigate the inherent cytotoxicity of the enzyme, a split-DddA architecture was developed: the enzyme is divided into two inactive halves, each fused to the C-terminus of a TALE (Transcription Activator-Like Effector) domain. To enhance editing efficiency by suppressing DNA repair mechanisms, a Uracil Glycosylase Inhibitor (UGI) was appended to the N-terminal DddA half [154]. TALE-mitoDdCBE has demonstrated success not only in cell cultures but also in editing mitochondrial DNA in embryos, facilitating the generation of animal models with specific mtDNA mutations [155,156,157].

Subsequent research has led to the development of various DddAtox variants with improved precision and relaxed sequence-context requirements (beyond the 5′-TC motif). Mi et al. identified a DddA homolog from *Simiaoa sunii* capable of efficient deamination within the 5′-GC context, which was previously recalcitrant to editing [158]. Similarly, Sun et al. proposed DddAs from *Streptomyces* sp. BK438 and *Lachnospiraceae sunii* NSJ-8 for 5′-GC targets, and a DddA from *Ruminococcus* sp. AF17-6 for 5′-AC motifs [159]. Furthermore, an ortholog from *Burkholderia gladioli* (BgDddA) demonstrated higher editing frequencies at 5′-TC sites compared to the canonical DdCBE, and its fusion with the Rta transactivator significantly bolstered editing efficiency at non-5′-TC targets [159]. By 2024, Castillo et al. achieved context-independent editing, effectively neutralizing the influence of the 5′-flanking nucleotide [160]. Additionally, Lee et al. (2023) successfully reduced off-target mutations through targeted amino acid substitutions within the DddA domain [161].

Barrera-Paez et al. employed DdCBE to address the m.5024C>T mutation in a C57BL/6N mt-tRNA(Ala) mouse line. Since DdCBE induces C-to-T transitions and can-not facilitate the reverse T-to-C correction, the authors utilized a creative approach: they introduced a compensatory mutation (m.5081G>A). This was designed to restore critical base-pairing within the mt-tRNA(Ala) molecule, mimicking natural evolutionary “rescue” events. For delivery, a dual-AAV system was used, with each DdCBE subunit packaged into a separate AAV9 vector under the control of a CMV promoter and administered systemically. While high-efficiency editing was observed in the heart, there was virtually no effect in the brain, kidneys, or liver. The lack of editing in the liver is particularly paradoxical given the high tropism of AAV serotypes for this organ; the authors attributed this to an unexplained failure in the synthesis of the TALE DNA-binding domain in hepatic tissue. This phenomenon represents a significant hurdle for DdCBE applications and warrants further independent investigation [162].

A critical and relatively understudied risk associated with DdCBE is the accelerated generation of NUMTs (Nuclear mitochondrial DNA). NUMTs are fragments of mtDNA that have integrated into the nuclear genome, a process known as numtogenesis. This process occurs spontaneously throughout the human lifespan and is linked to the continuous translocation of mitochondrial material into the nucleus [163,164,165]. While often benign, NUMT insertions can cause genetic disorders, such as Pallister–Hall syndrome [166], severe Factor VII deficiency [167], and mucolipidosis type IV [168]. Recent evidence also highlights the role of NUMTs in oncogenesis [169,170,171]. Alarmingly, Wu et al. observed that the use of DdCBE can increase the frequency of NUMTogenesis 11-fold, posing a potential threat to genomic stability that must be addressed in future therapeutic developments. Intriguingly, although DdCBE-mediated editing does not involve direct DNA cleavage, the emergence of NUMTs is likely associated with mtDNA degradation and the subsequent release of fragmented mitochondrial DNA into the cytoplasm [172]. Nevertheless, when mitoTALEN—which functions specifically by inducing double-strand breaks—was targeted to the ND4 gene, the frequency of NUMTogenesis increased only 4-fold. This is nearly three times lower than the frequency observed with DdCBE. This paradoxical result is likely attributable to the instability of uracil-containing DNA generated during the DdCBE editing process [154]. To mitigate this issue, Wu et al. proposed the incorporation of exonucleases TREX1n or TREX2, which efficiently degrade linear DNA. These enzymes can be either fused directly to DdCBE or targeted to the mitochondria independently via an MTS; this strategy has been shown to reduce the probability of NUMT integration by more than 2-fold [173].

Commonly, these data suggest that while DdCBEs hold significant therapeutic potential, further refinements in precision and the development of robust dose-control strategies are required before clinical translation. In a related development, Yin et al. have reported the use of DddB for cytosine deamination in dsDNA, which exhibits sub-stantially higher activity toward non-5′-TC targets compared to the canonical DddA [174].

Shortly after the discovery of mitoDdCBE, an A-to-G mitochondrial base editor named TALED was introduced [175]. The TALED architecture is broadly similar to DdCBE, incorporating TALE domains, an MTS, and BcDddA in either full-length or split forms. However, it replaces the UGI with TadA8e—an adenine deaminase engineered from the *E. coli* TadA enzyme that induces A-to-G transitions in single-stranded DNA (ssDNA) [176,177].

Based on these components, three distinct TALED configurations have been developed: monomeric TALEDs (mTALEDs), consisting of a single TALE array fused to TadA8e, followed by a full-length BcDddA variant; dimeric TALEDs (dTALEDs), where BcDddA and TadA8e integrated into separate TALE arrays; split TALEDs (sTALEDs), which utilizes G1397-split BcDddA variants combined with separate TALE arrays, where the deaminase domains follow the split segments. These three architectures exhibit different preferences for adenosine editing positions and distinct shifts in their respective editing windows [175]. TALEDs have demonstrated successful editing at 17 human mitochondrial loci, with frequencies reaching up to 49% [175].

TadA8e, much like its natural counterpart TadA, exhibits high off-target activity on both DNA and RNA. Interestingly, TadA8e showed a much greater propensity for inducing RNA mutations when directed to the nucleus via a nuclear localization signal (NLS) than when targeted to the mitochondria via an MTS. Nevertheless, the introduction of specific point mutations allowed for the development of variants with editing precision exceeding 99%. Using the mutant sTALED-V28R variant, researchers successfully generated mice carrying mitochondrial mutations m.8585T>C and m.8591T>C (corresponding to human mutations m.T9185C and m.T9191C). The reported mutation frequency was approximately 20%, with off-target mitochondrial mutations limited to ~1% and no detectable nuclear off-target effects [178]. To further reduce cellular toxicity, it was found necessary to incorporate the E1347A amino acid substitution in BcDddA. Additionally, Wang et al. recently reported the creation of mtDNA-mutant rabbit models using sTALEDs in embryos [179].

Both DdCBE and TALED perform base deamination on both strands of double-stranded DNA (dsDNA) within the editing window. However, single TALE tethering can lead to unintended effects in split-DddA systems [180]. Since the deamination of neither cytosine nor adenine should occur in a base-paired state [181], it was hypothesized that inducing single-stranded DNA (ssDNA) exposure around the target loci could facilitate more efficient and targeted mitochondrial deamination. Consequently, numerous research groups have focused on developing base editors that target single-stranded DNA (ssDNA). To this end, methods for efficient and precise mtDNA base editing have been developed by combining deaminases with nickases. In 2025, Zhang et al. utilized the SsdA deaminase from *Pseudomonas syringae* for C-to-T base editing. SsdA is a deaminase toxin that specifically acts on ssDNA. When fused with nCas9-UGI, SsdA demonstrated successful nuclear genome editing in 293T and K562 cells [182]. Subsequently, Kweon et al. employed SsdA targeted to four mitochondrial genome loci via TALE domains. Although its efficiency is currently lower than that of TALE-DdCBE, its monomeric nature—contrasting with the dimeric DdCBE architecture—and potential for optimization make it a promising candidate for further development. Interestingly, TALE-SsdA showed low but detectable mitochondrial editing (up to 5%), while remaining completely ineffective in the nucleus. However, since the specific targeting signals and localization data were not provided, suboptimal organelle targeting remains a plausible explanation for this low efficiency [183].

Hu et al. developed CyDENT, a platform comprising a pair of TALE domains fused to a FokI nickase, an ssDNA-specific cytidine deaminase (such as Sdd7 or Sdd3), and an exonuclease to generate the ssDNA substrate required for deamination. Specifically, the Trex2 exonuclease was fused to the N-terminus of a TALE array linked to a FokI nickase domain to facilitate nucleotide cleavage around the nick site, while the ssDNA-specific cytosine deaminases were fused in a similar position on the partner TALE array. Additionally, UGIs were linked to the C-terminus of FokI-L and the N-terminus of FokI-R, respectively. The average editing frequency of mtCyDENT across seven endogenous mtDNA sites in HEK293T cells ranged from 1.16% to 11.7%. Notably, the incorporation of a viral RNA silencing suppressor peptide γb (generating the mtCyDENT1b variant) further increased the average editing efficiency by 2.42–6.18-fold, reaching levels of 4.55–39.3% [184]. Another research group developed mitoBes (mitochondrial base editors) by combining various nickases and deaminases. In this strategy, one TALE array is fused to a nickase that generates a transient ssDNA region without the requirement for an exonuclease. Meanwhile, the second TALE array, fused to an ssDNA deaminase, mediates base editing on the exposed strand. To enhance efficiency and precision, several highly efficient nickases were evaluated, including MutH (5′-↓GATC-3′), MutH* (E91A, F94A) (5′-↓GAT-3′), and Nt.BspD6I(C). MutH functions as a sequence-specific nickase recognizing the 5′-GATC motif, with its preferences shaped by both the structural characteristics of the target sequence and the spatial distance between the TALE binding site and the motif [185]. The MutH variant, containing E91A and F94A mutations, significantly expanded the targetable range by recognizing a broader 5′-GATN motif [186]. In contrast, Nt.BspD6I(C), derived from the C-terminal cleavage domain of Nt.BspD6I [187], exhibited no sequence or context restrictions.

Leveraging highly efficient ssDNA deaminases—TadA8e-V106W for adenine and APOBEC1 for cytosine—new tools designated mitoABE and mitoCBE, respectively, were developed. These systems enable efficient and selective base editing in mitochondria. These systems exhibit a superior specificity profile, as the deaminases employed exhibit high activity exclusively on single-stranded DNA (ssDNA), with negligible or non-existent activity on double-stranded DNA. The specificity of dimeric mitoBEs is further bolstered by the requirement that both TALE-fusion proteins bind to their respective target sites; neither the TALE-deaminase nor the TALE-nickase can initiate base editing independently. Furthermore, mitoBEs demonstrate high strand-selectivity.

The editing window of dimeric mitoBEs can be precisely controlled, as it is defined by the two flanking TALE binding sequences. Researchers screened 70 homologous loci in mouse cells corresponding to pathogenic human mtDNA mutations, successfully editing 68 sites, with 8 sites achieving editing efficiencies of approximately 20%. Notably, no off-target effects were detected in either the mitochondrial or nuclear genomes. Building on these optimizations, preliminary analyses of these enhanced mitoBEs confirmed high editing efficiency (averaging ~20% in mtDNA) for at least 8 pathogenic point mutations in mouse models. Furthermore, this system was successfully employed to generate a mouse model harboring the MT-ND5 m.12784A>G mutation, which exhibited disease-relevant phenotypes. This study confirms the efficacy of this strategy for generating mitochondrial disease models and highlights the broad therapeutic potential of optimized mitoBEs, although clinical translation remains contingent on further validation of in vivo delivery and editing specificity for a wider spectrum of pathogenic mutations [186,188,189].

Remarkably, despite the high specificity for mtDNA, numerous reports indicate that mitoABE induces significant off-target effects throughout the transcriptome during the editing process. This finding underscores the necessity for further precision engineering of the base editor [190]. Based on structural optimizations of TALED, the TadA8e-V106W component in mitoABE was replaced with TadA8e-V106W-V28F.

While mitoCBE demonstrates higher editing efficiency compared to mitoABE, it also triggers increased off-target editing events within the mitochondrial genome. Crucially, this increase in off-target effects is independent of the TALE array specificity and is likely attributable to stochastic deamination mediated by rAPOBEC1 [191]. To resolve this issue, APOBEC1 was replaced with CBE6d—a cytidine deaminase derived from the TadA enzyme family [192]. This modification not only ensured robust editing efficiency but also narrowed the editing window and minimized mitochondrial off-target effects during cytosine base editing [191]. The various mitochondrial genome editing tools are listed in Table 4.

## 9. Conclusions

Although the drug based on allotopic expression of MT-ND4 for the treatment of LHON has successfully navigated clinical trials, it remains uncertain whether this approach will prove equally effective for other mitochondrial genes. A potential explanation for the success of MT-ND4 therapy relative to other targets lies in the fact that, in Leber Hereditary Optic Neuropathy (LHON), the therapeutic agent is administered directly via intravitreal injection. In contrast, most other mitochondrial DNA (mtDNA) mutations manifest with neurological symptoms necessitating brain targeting—a far more formidable challenge. However, rapidly advancing brain-targeting strategies, such as synthetic adeno-associated virus (AAV) serotypes with enhanced blood–brain barrier (BBB) permeability [193] and cerebrospinal fluid (CSF) delivery [194], may alter this landscape. A primary driver for allotopic expression is its fundamental advantage over genome editing: while editing tools require labor-intensive design for each specific mutation—and some mtDNA defects, such as large-scale deletions, remain refractory to current editing technologies—an optimized copy of the target gene can be utilized to address any functional impairment within that gene, regardless of the mutation type or location.

CRISPR-based editors, which initially sparked significant optimism [103], have demonstrated limited efficacy in mitochondria [106,137]. While recombination deficiency in mitochondria affects all editing platforms, the inefficient transport of RNA—often precluding successful sgRNA delivery—is a hurdle unique to CRISPR systems that other editing modalities circumvent. Nonetheless, RNA-guided targeting remains attractive due to its programmable simplicity. Emerging systems like Cas12 and Argonaute, which utilize shorter and (in the case of Argonaute) PAM-independent RNAs, may provide robust alternatives to traditional Cas9 [141]. Platforms utilizing programmable protein domains for DNA recognition predated CRISPR [195] but faced long-standing obstacles, such as complex design requirements and the necessity for dimeric subunits. The rise of CRISPR likely led to a temporary decline in interest in TALEN- and ZFN-based tools. However, traditional nuclease approaches have regained prominence with the recent development of compact, monomeric programmable nucleases like ARCUS [151]. Furthermore, advances in mitochondrial delivery methods may facilitate the entry of larger or poorly permeable components.

Another breakthrough in recent years has been the advent of base editors [154,178]. Although these tools encounter significant clinical translation barriers—notably off-target editing and strong context dependency [196]—the last five years have yielded impressive milestones, including the creation of animal models [155,178] and the correction of mutations in patient-derived cells [154]. Nevertheless, given their current low editing efficiency and high frequency of off-target events [154,178], base editors are not yet sufficiently precise for clinical applications. It is noteworthy, however, that these disruptive technologies have enabled the introduction of specific mtDNA mutations in model animals—a process that was previously arduous and time-consuming [190]. Consequently, they will significantly streamline the development and validation of future therapies while deepening our understanding of how these mutations influence systemic physiological processes.

In conclusion, allotopic expression of protein-coding genes represents the most promising near-term approach with the highest probability of clinical implementation in the coming years. Meanwhile, therapies based on programmable nucleases and base editors—technologies in which target DNA sequence recognition is performed via RNA-independent mechanisms—can induce double-strand breaks (DSBs) in mtDNA to drive heteroplasmy shifts or facilitate the precise correction of genetic defects, respectively. Clinical translation will necessitate the development of base editors that combine high precision and specificity, as well as programmable nucleases characterized by ease of targeting and efficient mitochondrial import.

## Figures and Tables

**Table 1:** Some of the most common mutations in the mitochondrial genome.

Mutation	Gene	Disease
m14484T>C	*MT-ND6*	LHON [10,11]
m.8344A>G	*MT-TK*	MERRF [12]
m.3243A>G	*MT-TL1*	MELAS [13,14]
m.8993T>G	*MT-ATP6*	LEIGH SYNDROME [15,16]
m.11778G>A	*MT-ND4*	LHON [17,18]
m.3460G>A	*MT-ND1*	LHON [19]
m.13513G>A	*MT-ND5*	MELAS [20,21]
m10197G>A	*MT-ND3*	Marsden syndrome [22]

**Table 3:** Expression of protein-coding genes (including allotopic and xenotopic approaches) for rescue the mitochondrial genome mutation.

Target Gene	Therapeutic Gene	Target Mutation	Reference
*MT-TV*	*VARS2* (*Homo sapiens*)	m.1624C>T	[86]
*MT-TH*	*HARS2* (*Homo sapiens*)	m.12201T>C	[87]
*MT-TA*	*AARS2* (*Homo sapiens*)	m.5655A>G	[88]
*MT-TL1*	*LARS2* (*Homo sapiens*)	m.3243A>G	[89]
*MT-TL1*	*EFTu*, *EFG2* (*Homo sapiens*)	m.3243A>G	[82]
*MT-TK*	*MTO1* (*Homo sapiens*)	m.8344A>G	[83]
*MT-TK*	*TRMT61B* (*Homo sapiens*)	m.8344A>G	[94]
*MT-ND1*	*NDI1* (*Saccharomyces cerevisiae*)	m.3460G>A	[68]
*MT-ND4*	Optimized *ND4* (*Homo sapiens*)	m.11778G>A	[27]
*MT-ATP6*, *MT-ATP8*	Optimized *ATP6* (*Homo sapiens*), Optimized *ATP8* (*Homo sapiens*)	m.8529G → A	[96]
*MT-ATP6*	Optimized *ATP6* (*Homo sapiens*)	8993T>G	[26]
*MT-ND3*	Optimized *ND3* (*Homo sapiens*)	m.10197G>C, m.10191T>C	[30]
*MT-ATP6*	*ATP6* (*Chlamydomonas reinhardtii*)	m.8993T>G	[66]
*MT-ND1*	Optimized *ND1* (*Homo sapiens*)	m.3460G>A	[27]

**Table 4:** Tools for editing the mitochondrial genome.

Tool	Mechanism of Action	Structure	Limitations	Advantages	References
MitoZFN	Elimination of specific mtDNA	Heterodi meric protein	Non-compact	RNA-free	[143,144]
MitoTALEN	Elimination of specific mtDNA	Heterodi meric protein	Non-compact	RNA-free, high-precision	[146,147,148]
MitoARCUS	Elimination of specific mtDNA	Monomeric protein	Targeting is limited	RNA-free, compact	[151]
AmAgo	Elimination of specific mtDNA	Monomeric protein + 18 bp RNA	RNA-dependent	Pam-independent, compact, easily programmable	[141]
CRISPR/Cas9	Elimination of specific mtDNA	Monomeric protein + 100 bp RNA	RNA-dependent	Easily programmable	[87,88,103,105]
CRISPR/Cas12a	Elimination of specific mtDNA	Monomeric protein + 40 bp RNA	RNA-dependent	Easily programmable	[139,140]
DdCBE	mtDNA point mutation (C-to-T)	Heterodimeric protein	Off-targeting effect, non-compact	High flexibility	[154,155]
TALED	mtDNA point mutation (A-to-G)	Heterodimeric or monomeric protein	Bystander editing	High flexibility	[175,176,177,178]
CyDENT	mtDNA point mutation (C-to-T)	Heterodimeric protein	Non-compact, low efficienty	High flexibility and accuracy	[184]
mitoBEs	mtDNA point mutation (C-to-T) or (A-to-G)	Heterodimeric protein	Non-compact	High flexibility and accuracy	[186,189]
SsdA	mtDNA point mutation (C-to-T)	Monomeric protein	Low efficiency	High flexibility and accuracy	[183]

## Data Availability

No new data were created or analyzed in this study. Data sharing is not applicable to this article.

## Abbreviations

The following abbreviations are used in this manuscript:

AAV	Adeno-associated virus
BBB	Blood–brain barrier
CNS	Central nervous system
CRISPR	Clustered Regularly Interspaced Short Palindromic Repeats
sgRNA	Single-guide RNA
MELAS	Mitochondrial encephalomyopathy with lactic acidosis and stroke-like episodes
MERRE	Myoclonic Epilepsy with Ragged Red Fibers
mtDNA	Mitochondrial DNA
MTS	Mitochondria targeting signal
PAM	Protospacer adjacent motif
NARP	Neurogenic muscle weakness, ataxia, and retinitis pigmentosa
lncRNA	Long non-coding RNA
LHON	Leber hereditary optic neuropathy
TALEN	Transcription Activator-Like Effector Nucleases
rAAV	Recombinant Adeno-associated virus
RIC	RNA Import Complex
OxPhos	Oxidative phosphorylation
ZFN	Zinc-finger nucleases
UTR	Untranslated Regions
